# Optimizing the Implementation of Tobacco Treatment for People with HIV: A Pilot Study

**DOI:** 10.3390/ijerph191912896

**Published:** 2022-10-08

**Authors:** Madeline G. Foster, Benjamin A. Toll, Emily Ware, Allison Ross Eckard, Katherine R. Sterba, Alana M. Rojewski

**Affiliations:** 1 Department of Public Health Sciences, Medical University of South Carolina, Charleston, SC 29425, USA; 2 Hollings Cancer Center, Charleston, SC 29425, USA; 3 Clinical Pharmacy, Medical University of South Carolina, Charleston, SC 29425, USA; 4 Departments of Pediatrics and Medicine, Divisions of Infectious Diseases, Medical University of South Carolina, Charleston, SC 29425, USA

**Keywords:** smoking cessation, tobacco treatment, implementation, HIV/AIDS

## Abstract

People with HIV (PWH) have higher rates of tobacco use compared to their societal counterparts and are disproportionately affected by tobacco-related morbidity and mortality. A needs assessment was conducted to assess provider beliefs and opinions on tobacco treatment barriers and treatment approaches. The results highlighted a disconnect between the known importance of quitting smoking and barriers in linking patients to treatment, such as lack of patient interest and other patient issues being a higher priority. Using this assessment data, a treatment delivery approach, Proactive Outreach with Medication Opt-out for Tobacco Treatment Engagement (PrOMOTE), was devised and piloted. PrOMOTE consisted of an outpatient clinical pharmacist trained in tobacco treatment proactively contacting patients for counseling and to prescribe smoking cessation pharmacotherapy (varenicline or dual nicotine replacement therapy (NRT)) using an opt-out approach. The pilot was conducted with 10 PWH and patient reach and opt-out rates were evaluated. Of the 10 patients contacted, 7 were reached and none opted out of the pharmacotherapy prescription (varenicline = 6; NRT = 1). Providers know the importance of smoking cessation for PWH but encounter several barriers to implementing treatment. Using PrOMOTE methods to deliver tobacco treatment increased the reach and pharmacotherapy acceptance rate of PWH who smoke.

## 1. Introduction

Since the development of antiretroviral therapy (ART), the survival rates of people with HIV (PWH) have continually improved [1], and their life expectancy is now nearing that of the general population (nearly 70 years of age) [2]. Despite the survival benefits of ART, PWH continue to face significant impacts regarding morbidity and mortality due to other modifiable risk factors. The leading cause of cancer death among PWH is lung cancer [3], and cigarette smoking contributes to this health crisis. Among PWH, tobacco use rates are 40–50% [4], nearly triple the 15.2% rate of combustible tobacco product use in the general U.S. population [5]. Prior research has shown that approximately 50% of PWH are interested in quitting and 89% have made a quit attempt once in their lifetime [6] but have difficulty achieving abstinence [7]. PWH who continue to smoke after their diagnosis are approximately 20% more likely to die from lung cancer than their societal counterparts [3]. Developing effective smoking cessation interventions for PWH and connecting this population to treatment is imperative to reduce the risk of morbidity and mortality from a smoking-related disease.

Clinical practice guidelines for treating tobacco use indicate that evidence-based pharmacological and behavioral interventions are recommended for all individuals who use tobacco, including PWH [8,9]. However, prior research has shown that smoking is often not addressed in the context of HIV care and few PWH are offered tobacco treatment. An evaluation of HIV Medical Association providers’ beliefs and practices [10], found that providers agreed that smoking is an important issue in PWH, but less than half agreed that they frequently prescribed varenicline and nicotine replacement therapy (NRT) despite the safety and efficacy demonstrated with these first-line pharmacotherapies in PWH [11,12]. In addition, a variety of barriers complicate evidence-based treatment implementation such as insufficient training, lack of therapeutic support, and limited time by providers [9,13].

With several effective tobacco treatment approaches at a patient’s disposal, optimizing how those treatments are implemented and delivered to PWH requires an empirical approach. We conducted a needs assessment to better understand the status of tobacco treatment implementation and common barriers to reach PWH at an infectious diseases (ID) clinic within an academic medical center. Using this assessment data, a treatment delivery approach was devised and piloted.

## 2. Needs Assessment

### 2.1. Needs Assessment Methods

Tobacco treatment is currently managed in this ID clinic by a clinical pharmacist using a reactive, opt-in approach. In routine clinic appointments, the clinical pharmacist is tasked with coordinating pharmacotherapy needs (e.g., diabetes management, smoking cessation, etc.). If a patient currently smokes and indicates interest in quitting, the clinical pharmacist will inquire about smoking cessation pharmacotherapy preferences, and prescriptions are provided if the patient opts in. The ID physician may also refer the patient to the clinical pharmacist if they request assistance with quitting smoking.

A perceived barriers instrument was previously developed to guide practice change interventions in the healthcare setting guided by the implementation of science principles [14]. The original instrument was adapted by two of the authors (AMR and KRS) with the addition of several items addressing barriers in the clinical tobacco treatment context. The survey was distributed via email to all providers within the ID clinic to ascertain: a) perceived importance of quitting smoking in PWH (0 = Not at All Important to 4 = Extremely Important), b) confidence that existing options were successfully reaching PWH in the clinic (0 = Not at All Confident to 4 = Extremely Confident), c) barriers for delivering treatment to PWH (1 = Not at All a Barrier to 4 = Major Barrier), d) confidence in communication about smoking and treatment (0 = Not at All Confident to 4 = Extremely Confident), and e) perceived importance of treatment modalities (0 = Not at All Important to 4 = Extremely Important).

### 2.2. Needs Assessment Results

Twelve providers responded to the survey, and their roles in the clinic were physician (5), social worker (3), program assistant (1), certified medical assistant (1), pharmacist (1), and resident (1). All respondents reported that it was “very/extremely important” for PWH to quit smoking; although, the majority (83.3%) were only moderately or less confident that the existing tobacco treatment options were successfully reaching patients. As highlighted in Table 1, the most frequently endorsed major or moderate barriers to tobacco treatment among PWH included lack of patient interest, other patient issues being a higher priority, time, and patient financial challenges. The majority had high confidence in communicating about the consequences of smoking and the benefits of cessation, yet had lower confidence in other areas such as providing patient-centered tobacco treatment recommendations. Finally, the majority of respondents perceived meetings with patients, pharmacotherapy, and an efficient communication system for multi-disciplinary team members as important components of successful tobacco treatment interventions for PWH.

## 3. Pilot Study

### 3.1. Pilot Study Methods

The needs assessment results guided a new intervention that consisted of remote contact, pharmacotherapy, 3 scheduled counseling sessions, and clinical encounter documentation posted to the patient’s chart to keep multi-disciplinary team members informed about tobacco treatment efforts. These components, combined with a proactive [15,16] and opt-out [17,18,19] approach, formed the basis of Proactive Outreach with Medication Opt-out for Tobacco Treatment Engagement (PrOMOTE) to optimize tobacco treatment delivery in the ID clinic.

A pilot evaluation of PrOMOTE was conducted to assess the feasibility of the approach. Given that this was a quality improvement project and an extension of clinical care, formal patient consent was not obtained. Ten PWH were selected from the clinic based on current smoking status and were contacted via telephone by a clinical pharmacist at the MUSC Tobacco Treatment Program. The clinical pharmacist conducted a brief (40 min) motivational interview and counseling session, which was based on practical counseling, a cognitive-behavioral smoking cessation treatment modality [8].

The clinical pharmacist reviewed the patient’s chart to ensure that the individual qualified for varenicline and/or NRT (e.g., based on past adverse reactions to either medication, patient comorbidities, etc.). Preference was given to prescribing varenicline over NRT [9,11,12,20,21]. The opt-out language from the clinical pharmacist was scripted: “What I would like to do is prescribe you varenicline to help you quit smoking. I have reviewed your chart and varenicline is safe to use with your other medications and has been shown to reduce cravings to smoke. I reviewed your prescription coverage, and this medication will be $_____. If I can verify your address, I will mail this directly to you.” Patients could elect to pick up the medication from a pharmacy instead of using the mail-order pharmacy services. If the patient opted out of varenicline use or varenicline was contraindicated, the pharmacist offered dual NRT (transdermal patch and oral gum/lozenge) or single NRT if indicated. If the patient opted out of all medication use, the pharmacist conducted a brief motivational interview to encourage the use of smoking cessation pharmacotherapy. If the patient still opted out, the pharmacist offered to follow up for behavioral counseling.

All patients were encouraged to start their medication upon receipt and to set a quit date 1 week from medication initiation. The varenicline prescription followed the standard titration schedule: 0.5 mg once per day for days 1–3, 0.5 mg twice per day for days 4–7, then 1 mg twice per day. The dual NRT (or single NRT) prescription followed the standard dosing recommendations: daily 21 mg patch for those smoking ≥10 cigarettes per day (CPD) and a 14 mg patch for those smoking <10 CPD, and 4 mg mini lozenge if they smoke < 30 min of waking or 2 mg mini lozenge if they smoke after more than 30 min of waking, taking 9 to 20 lozenges per day. Pharmacotherapy switching (from varenicline to NRT or vice versa) was permitted if the patient reported treatment failure or a high frequency of side effects after 1 week of appropriate use based on the clinical judgment of the pharmacist. Pharmacotherapy was covered by the patient’s insurance, which may have consisted of private insurance and/or the Ryan White/AIDS Drug Assistance Program. Patients remained on pharmacotherapy for 12 weeks total. The pharmacist scheduled two additional appointments with patients approximately 3–4 weeks apart to assess smoking status, conduct additional counseling (approximately 20 min), and assess medication use and side effects. Follow-up contact was attempted 5 times. Contact was attempted more than once for each time point if there was no response via MyChart, postal mail, and phone to attempt re-engagement.

### 3.2. Pilot Study Results

The summary of individual patients and their prescription status is presented in Table 2. On average, the patients were 50 years of age, 70% male, 60% African American/Black, and the modal number of cigarettes smoked per day was 10 (range: 2–40 CPD). Of the 10 patients contacted, there was a 70% reach rate. Of the seven patients reached, six accepted a varenicline prescription, one continued their pre-existing prescription for bupropion and the pharmacist prescribed a nicotine patch in addition, and none opted out. Of the six patients who were prescribed varenicline, five received their prescription. Follow-up data were available from chart review for five of the seven patients who had been initially reached for treatment. Three patients self-reported a reduction in cigarettes per day by at least half of their baseline smoking rate, and 2 self-reported that they were abstinent.

## 4. Discussion

The needs assessment highlighted a disconnect between the known importance of smoking cessation and the challenge in linking patients to treatment due to a lack of time on the part of the provider and less confidence about how to facilitate a tobacco quit attempt. ID clinics are tasked with many components of care for PWH: outpatient medical care, medical case management, mental health services, reproductive health services, outpatient substance abuse services, treatment adherence counseling, and psychosocial support services. Indeed, two of the most frequently provider-endorsed major or moderate barriers to tobacco treatment among PWH included patient issues being a higher priority and lack of provider time. All providers viewed quitting smoking as important, but this objective may ultimately become a lower priority in an environment in which multimorbidity is common and patient care is complex. Another frequently endorsed barrier to tobacco treatment was the lack of patient interest. When the onus of responsibility is on the patient to opt in to treatment and motivation to quit smoking may fluctuate, patients may be less likely to pursue tobacco treatment on their own [22].

The pilot study demonstrated preliminary feasibility and acceptability for a proactive, opt-out approach delivered by a tobacco treatment specialist-trained clinical pharmacist. The outreach was acceptable as the provider achieved a 70% reach rate and a 60% acceptance of pharmacotherapy. This finding highlights that presenting tobacco treatment in an opt-out fashion may increase reach and engagement with first-line pharmacotherapy options. Even though the counseling component was not provided in the context of the ID clinic, the remote nature of the provider outreach did not increase the patient burden for accessing care. This preliminary demonstration suggests that the PrOMOTE intervention may help to optimize tobacco treatment delivery to PWH.

This pilot study had several limitations. There was no control group, but a planned trial (ClinicalTrials.gov: NCT05019495) will randomize participants and compare the effectiveness of this approach to the current standard of care in the ID clinic. The smoking status of patients was also assessed by self-report, and future studies should employ a biochemical verification of smoking status. The goals of the pilot study were to assess the feasibility and acceptability of the PrOMOTE intervention; thus, the abstinence outcome data for these patients are not presented. Future studies will evaluate abstinence rates in addition to reach rates.

## 5. Conclusions

Given the high rates of mortality and morbidity among PWH who smoke [3,23,24], creative and rigorous approaches are desperately needed to overcome barriers to tobacco treatment implementation in this important high-risk population. This promising PrOMOTE intervention warrants additional evaluation on a larger scale coupled with an assessment of pharmacotherapy utilization, tobacco cessation outcomes (including quit attempts), and additional implementation processes.

## Figures and Tables

**Table 1:** Needs assessment results.

Perceived Barriers to Tobacco Treatment in ID	% Endorsing Moderate/Major Barrier
Lack of patient interest	92%
b. Other patient issues are higher priority	83%
c. Financial challenges for patients	58%
d. Not enough time	58%
e. Lack of designated staff	33%
f. Lack of provider reimbursement	33%
g. Unsure of resources available	33%
h. Lack of provider training	25%
i. Pharmacotherapy is incompatible with ART	17%
j. Lack of evidence for PWH interventions	8%
k. Lack of systemic process for documenting smoking status	8%
Confidence in Communicating Aspects of Tobacco Treatment	% Very/Extremely Confident
Benefits of quitting smoking	50%
b. Consequences of continued smoking	42%
c. Eliciting patient goals with respect to quitting smoking	25%
d. Motivating a patient to quit	25%
e. Providing patient-centered tobacco treatment recommendations	8%
Importance of Strategies in Tobacco Treatment for PWH	% Very/Extremely Important
Facilitating pharmacotherapy use	75%
b. Meeting with patients, remotely	67%
c. Routine telephone calls to patients, with counseling	67%
d. Meeting with patients, in-person	58%
e. Efficient communication system for multi-disciplinary teams	50%
f. Provision of list of resources	17%

**Table 2:** Pilot Results.

Patient	Initial Contact (Y/N)	Baseline CPD	Prescription	Prescription Received (Y/N)	Follow-Up CPD
1	Y	20	Varenicline	N	Lost to follow-Up
2	Y	40	Varenicline	Y	20
3	Y	20	Nicotine Patches *	Y	0
4	N	10	--	--	--
5	Y	10	Varenicline	Y	3
6	N	2	--	--	--
7	Y	5	Varenicline	Y	Lost to follow-up
8	Y	10	Varenicline †	Y	0
9	Y	30	Varenicline †	Y	<10
10	N	10	--	--	--

Note: * patient already taking bupropion; † patient already using 21 mg nicotine patch; CPD = cigarettes per day; Follow-up CPD was derived from chart review at most recent clinical follow-up.

## Data Availability

De-identified data from this study are not available in a public archive. De-identified data from this study will be made available (as allowable according to institutional IRB standards) by emailing the corresponding author.
